# VHH-Functionalized Silica-Coated Magnetic Nanoparticles for Immunoaffinity Isolation of Extracellular Vesicles

**DOI:** 10.3390/membranes16100327

**Published:** 2026-09-29

**Authors:** Jovana Terzić, Lidija Filipović, Ninoslav Mitić, Sanja Stevanović, Ario de Marco, Milica Popović

**Affiliations:** 1 Institute of Chemistry, Technology and Metallurgy, National Institute of the Republic of Serbia, University of Belgrade, 11000 Belgrade, Serbia; jterzic@chem.bg.ac.rs (J.T.); sanjas@ihtm.bg.ac.rs (S.S.); 2 Innovation Center of the Faculty of Chemistry, University of Belgrade, 11158 Belgrade, Serbia; filipoviclidija29@gmail.com; 3 Institute for the Application of Nuclear Energy (INEP), University of Belgrade, 11080 Belgrade, Serbia; ninoslavm@inep.co.rs; 4 Laboratory for Environmental and Life Sciences, University of Nova Gorica, 5000 Nova Gorica, Slovenia; ario.demarco@ung.si; 5 Faculty of Chemistry, University of Belgrade, 11158 Belgrade, Serbia

**Keywords:** extracellular vesicles, VHH, nanobodies, magnetic nanoparticles, immunoaffinity isolation, human plasma

## Abstract

Extracellular vesicles (EVs) are membrane-enclosed nanoparticles involved in intercellular communication and numerous physiological and pathological processes. Their molecular cargo reflects the state of the cell of origin, making EVs promising sources of biomarkers and potential therapeutic agents. However, efficient and selective isolation of EVs from complex biological fluids remains challenging. Immunoaffinity-based approaches offer high selectivity through the recognition of EV-associated surface markers by specific affinity ligands. In this study, silica-coated magnetite nanoparticles were functionalized with a mixture of five VHH–eGFP constructs and evaluated as a solid phase for immunoaffinity isolation of EVs from human plasma. Surface modification of the nanoparticles was confirmed by FTIR spectroscopy, while protein-binding studies showed a maximum binding capacity (*Qmax*) of 118.2 mg/g. The developed material was then applied for EV isolation from plasma, and the resulting EV-enriched preparations were characterized by protein and lipid quantification, nanoparticle tracking analysis, flow cytometry, and atomic force microscopy. NTA revealed a median particle diameter of 124 nm and a particle concentration of 6.9 × 10^9^ particles/mL. The presence of the EV-associated markers CD9, CD63, and CD81, together with their reduced signal following Triton X-100 treatment, supported the vesicular nature of the isolated particles. The affinity material could be reused over five consecutive isolation cycles, and EVs could be detected from plasma volumes as low as 25 µL. These results demonstrate the potential of VHH-functionalized magnetic nanoparticles as a reusable and adaptable platform for immunoaffinity-based EV isolation from human plasma.

## 1. Introduction

Extracellular vesicles (EVs) are membrane-enclosed particles released by a wide variety of cell types, with a molecular composition that reflects their cellular origin and physiological state. By transporting proteins, lipids, nucleic acids, and other biomolecules to recipient cells, EVs contribute to intercellular communication [1]. EVs can regulate many biological processes such as immune response, angiogenesis, tissue repair and cell proliferation [2]. EVs are present in a wide range of body fluids, including plasma, urine, saliva, cerebrospinal fluid, and breast milk [3,4]. EVs can be classified according to their biogenesis and size, with exosomes, microvesicles, and apoptotic bodies representing the three major biogenesis-based subtypes [5]. Exosomes are derived from the endosomal pathway and are generally 30–150 nm in size. Microvesicles are released by outward budding of the plasma membrane, and usually, their size range is between 50–1000 nm [6]. Apoptotic bodies are generated during apoptosis and are usually larger than other EV subtypes [7]. EVs have been considered with great interest as possible biomarkers for disease detection, monitoring and therapeutic management due to their stability in bodily fluids and their capacity to mirror the molecular composition of their cells of origin [8,9]. Furthermore, they have been considered as therapeutic agents and as carriers for therapeutic agents and drug-delivery vesicles. The growing biomedical potential has led to an increasing demand for reliable and efficient methods of isolation. However, the heterogeneity of EV populations and the complexity of the source biological fluids remain major obstacles to obtaining EV preparations of sufficient purity and reproducibility [10,11]. The isolation methods should provide sufficient purity for downstream analysis, good yields and preserve EV structural and functional integrity [12]. Common approaches for EV purification comprise differential ultracentrifugation, ultrafiltration, size-exclusion chromatography, polymer-based precipitation, and affinity-based techniques [13]. However conventional antibodies can present several drawbacks: high production costs, lengthy development timelines, batch-to-batch variability, and limited stability. Single-domain antibodies (such as VHHs) represent an attractive class of affinity ligands for EV isolations. Their small size, high stability, ease of production, and target specificity make VHHs suitable capture ligands for affinity-based EV isolation platforms. 

The VHH nanobodies used in this study were previously isolated from a naïve VHH library through biopanning against cell culture-derived EVs [14]. After immobilization on a solid substrate, they allowed the selective enrichment of EVs from different biological sources [15]. The performance of affinity-based EVs isolations strongly depends on the choice of the solid phase, as its physicochemical properties and compatibility with the recognition molecules can substantially affect yields, selectivity, and reproducibility of EV isolation.

Magnetic nanoparticles have attracted considerable attention as solid supports owing to their physicochemical properties [16,17]. Their relatively low toxicity, combined with the possibility of rapid and straightforward recovery from complex biological matrices using an external magnetic field, makes them particularly attractive for bioseparation applications. Their high surface-to-volume ratio also provides numerous accessible sites for the attachment of biomolecules [18]. These characteristics have enabled the widespread use of magnetic nanoparticles in biotechnology and biomedicine, including biomolecule separation, biosensing, drug delivery, diagnostics, and magnetic resonance imaging [19]. Among the various magnetic materials, the iron oxide magnetite (Fe_3_O_4_) is particularly attractive because of its favorable biocompatibility and magnetic properties [20,21,22]. Magnetic (Fe_3_O_4_) nanoparticles are widely used for magnetic separation because appropriately sized Fe_3_O_4_ nanoparticles can exhibit superparamagnetic behavior, facilitating magnetic recovery while reducing remanent magnetization after removal of external field. Fe_3_O_4_ nanoparticles can be synthesized using several approaches, including co-precipitation, thermal decomposition, hydrothermal synthesis, and solvothermal synthesis. Among these, co-precipitation is preferred because of its simplicity and relatively low cost [20]. However, since the bare Fe_3_O_4_ nanoparticles are easily oxidized and aggregated, which can affect their stability and limit their practical application [23,24]. Due to that fact they are often coated with silica to improve physicochemical stability and enable further surface modification, such as immobilization of biomolecules [25,26,27]. Consequently, silica-coated magnetic nanoparticles represent a particularly suitable solid support for immunoaffinity-based separation systems [28]. In this study, VHH nanobodies were immobilized on such nanoparticles to establish a stable and functional platform for the selective immunoaffinity isolation of EVs from human plasma.

## 2. Materials and Methods

### 2.1. Chemicals

Tetraethyl orthosilicate (TEOS, ≥99%) and (3-aminopropyl) triethoxysilane (APTES, ≥99%) were purchased from Thermo Fisher Scientific (Waltham, MA, USA). Iron(III) chloride (FeCl_3_), iron(II) sulfate heptahydrate (FeSO_4_·7H_2_O) and sodium hydroxide were of analytical grade (p.a.). Glutaric dialdehyde (25% *w*/*v*), fetal bovine serum and bovine serum albumin (BSA) were from SigmaAldrich (Steinheim, Germany). Anti-CD9 Phycoerythrin (PE)-labeled (clone MM2/57), Alexa Fluor^®^ 488 anti-human CD63 (clone H5C6) antibody and PE/Dazzle™ 594 anti-human CD81 (TAPA-1) antibody (clone 5A6) were from Bio-legend (San Diego, CA, USA). All other chemicals were p.a.

### 2.2. Synthesis of Magnetite Particles (MnPs)

**Figure 1:**
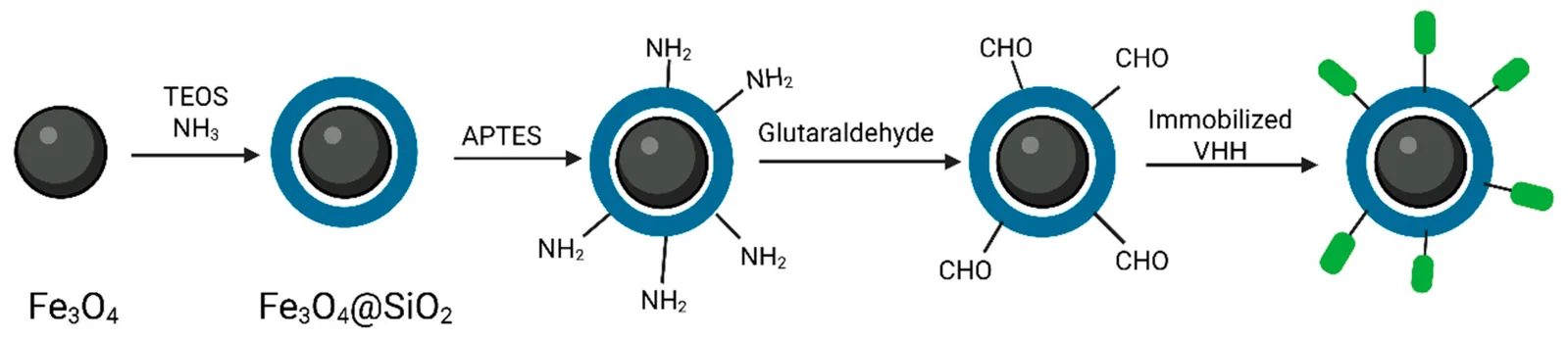
Schematic representation of the synthesis, surface functionalization and VHH immobilization on silica-coated magnetite nanoparticles.

Magnetite particles (Fe_3_O_4_) were prepared by coprecipitation of iron ions. Two precursor solutions were prepared by dissolving 0.324 g of anhydrous iron(III)-chloride (FeCl_3_) in 100 mL of deionized water and 0.278 g of iron(II)-sulfate heptahydrate (FeSO_4_ x 7H_2_O) in 100 mL deionized water [29]. The solutions were stirred and added with constant magnetic stirring to 100 mL of sodium hydroxide (NaOH) solution (0.1 M). The reaction mixture was further agitated at room temperature for 1 h to finish the production of magnetite particles. At the end of the reaction, the particles were separated and washed with deionized water to remove residual reactants and then with ethanol. Magnetite particles produced were coated with a silica layer using the Stöber technique [30]. The reaction mixture consisted of 60 mL ethanol, 15 mL deionized water and 1.5 mL ammonia. The mixture was homogenized and a suspension of 100 mg of magnetite particles in ethanol was added. A solution of tetraethyl-orthosilicate (TEOS) was prepared by mixing 0.6 mL TEOS with 1 mL ethanol. The reaction mixture was stirred for 4 h at room temperature during which a silicate shell was formed around the magnetite particles. Upon completion of the reaction, the silica-coated magnetite particles were separated by means of a magnet and washed three times with ethanol to remove excess unreacted TEOS. To functionalize the surface of silica-coated magnetite particles with amino groups, 350 µL of 3-aminopropyltriethoxysilane (APTES) was added to the particle suspension in 30 mL of ethanol [29]. The reaction mixture was stirred at room temperature for 2 h. After the end of the procedure, the amino-functionalized magnetite particles were separated by means of a magnet, washed three times with ethanol to remove excess APTES and resuspended in ethanol for further functionalization. The synthesis and functionalization of silica-coated magnetite nanoparticles are illustrated in Figure 1.

### 2.3. Production of VHH-eGFP

Five VHH–eGFP constructs (H1, H6, D5, B1, and G2), previously obtained by screening a naïve pre-immune VHH library against EVs derived from cell culture supernatants, were used in this study [14]. The corresponding VHH sequences were inserted between the *Nco*I and *Not*I restriction sites of a modified pET-14b vector [31], generating fusion proteins containing C-terminal eGFP and 6×His tags. Protein expression was performed in *Escherichia coli* BL21(DE3) cells co-expressing sulfhydryl oxidase and DsbC to support correct protein folding. VHH-eGFP production followed previously reported procedures with minor modifications [14,32]. For protein production, 400 mL of LB medium with 100 μg/mL ampicillin and 25 μg/mL chloramphenicol were inoculated with 1 mL of an overnight bacterial culture and grown at 37 °C until the OD_600_ reached the value of 0.4. Sulfhydryl oxidase and DsbC expression was initiated by adding 0.2% arabinose and lowering the cultivation temperature to 30 °C. After 30 min, VHH-eGFP expression was induced with 0.2 mM IPTG, followed by overnight cultivation at 21 °C. The harvested cells were resuspended in 20 mL of TBS (50 mM Tris-HCl, 500 mM NaCl, 5 mM MgCl_2_, pH 7.4), disrupted by sonication, and centrifuged at 12,000× *g* for 20 min at 4 °C. VHH-eGFP fusion proteins were purified from the resulting supernatant by immobilized metal affinity chromatography (IMAC). Before sample application, the resin was equilibrated with buffer A (50 mM Tris, 500 mM NaCl, 30 mM imidazole, pH 8.0). Unbound proteins were removed by washing with buffer A, while the VHH fusion proteins were recovered using buffer B (50 mM Tris, 200 mM NaCl, 300 mM imidazole, pH 8.0). Fractions containing purified VHH–eGFP were collected, supplemented with 30% glycerol, and stored at −20 °C until further use.

### 2.4. Scanning Electron Microscopy (SEM)

The morphology of the powdered magnetic particles was examined by field-emission scanning electron microscopy (FE-SEM) using a TESCAN MIRA3 microscope operated at an accelerating voltage of 20 kV. Prior to imaging, the samples were sputter-coated with Au–Pd using a Polaron SC502 sputter coater.

### 2.5. Fourier-Transform Infrared Spectroscopy (FTIR)

Fourier-transform infrared (FTIR) spectra of magnetite nanoparticles at different stages of surface modification were recorded using a Thermo Scientific™ Nicolet™ Summit™ X FTIR Spectrometer (Thermo Fisher Scientific, USA) equipped with an attenuated total reflectance (ATR) accessory. The spectra were recorded in ATR mode over the range of 4000–400 cm^−1^.

### 2.6. Determination of Static Binding Capacity

The static binding capacity of aldehyde-functionalized magnetite nanoparticles was assessed using BSA as a model protein. Five milligrams of nanoparticles were incubated with BSA solutions at concentrations ranging from 0.1 to 3 mg/mL in a batch system. The protein concentration in the supernatant was determined after incubation of 24 h using the Bradford Protein assay. All measurements were performed in duplicate. The binding capacity (mg bound protein per g dry nanoparticles) was calculated by subtracting the initial and equilibrium protein concentrations and was plotted against the equilibrium protein concentration to obtain the Langmuir binding isotherm. The experimental data were fitted to the Langmuir adsorption model using the following equation:

$$q=\frac{\mathrm{Qmax}*\mathrm{Ce}}{\mathrm{Kd}+\mathrm{Ce}}$$

where *Qmax* is the maximum binding capacity, *q* is the binding capacity, *Ce* is the equilibrium protein concentration, and *Kd* is the equilibrium dissociation constant.

### 2.7. Immobilization of VHH-eGFP on the MnPs (VHH-MnPs)

Amino-functionalized magnetite nanoparticles were activated by incubation with 2.5% glutaraldehyde in phosphate-buffered saline (PBS) for 2 h at room temperature with gentle stirring (Figure 1). After activation, the particles were magnetically collected and rinsed with PBS to eliminate residual glutaraldehyde. For VHH functionalization, 10 mg of activated nanoparticles were combined with 500 µg of VHH–eGFP, comprising 100 µg of each of the five VHH constructs, and incubated overnight at 4 °C with gentle mixing. The amount of unbound protein remaining in the supernatant after incubation was determined by Bradford assay to indirectly assess immobilization efficiency. The resulting VHH-functionalized magnetite nanoparticles were then used as the solid phase for the EV immunoaffinity isolation.

### 2.8. Blood Collection and Plasma Preparation

Human blood samples were obtained from healthy volunteers after written informed consent, in accordance with the Declaration of Helsinki and with approval from the Ethics Committee of the Faculty of Chemistry, University of Belgrade (Approval No. 2-6/24). Peripheral venous blood was collected into sodium citrate Vacutainer tubes and processed within 30 min of collection. Blood cells were removed by centrifuging whole blood at 1550× *g* for 30 min at room temperature, followed by centrifugation at 3200× *g* for 30 min to prepare platelet-free plasma (PFP). PFP was immediately used to isolate EVs or aliquoted and stored at −80 °C until further use.

### 2.9. EV Isolation via Magnetite Particle-Based Immunoaffinity Chromatography

VHH-functionalized magnetite nanoparticles containing 500 µg of immobilized VHH-eGFP per 10 mg of particles were used for EV isolation. The VHH-MNPs were incubated with 200 mM glycine (pH 7.0) for 30 min and subsequently with 5% (*w*/*v*) skim milk in PBS for an additional 30 min. After PBS washing, platelet-free plasma (PFP) from a single healthy donor was diluted 1:3 with PBS to a total volume of 1 mL and mixed with the VHH-MNPs for 1 h. The particles were then washed with PBS to remove unbound material. Captured EVs were recovered by two sequential elution steps, each performed with 400 µL of glycine buffer (pH 2.0). Each 400 µL eluate was immediately neutralized by adding 100 µL of Tris-HCl buffer (pH 9.1). The neutralized eluates were pooled to obtain a final EV isolate volume of 1 mL and stored at −20 °C until further characterization. For the negative isolation control, silica/APTES/glutaraldehyde-functionalized nanoparticles without immobilized VHH–eGFP were subjected to the same isolation procedure. The resulting control eluate was analyzed for protein content using the Bradford assay.

### 2.10. Nanoparticle Tracking Analysis (NTA)

The size distribution and concentration of isolated EVs were analyzed with a ZetaView^®^ Quatt PMX-430 instrument using ZetaView software version 8.05.16 SP3 (Particle Metrix, Inning am Ammersee, Germany). Before sample measurements, instrument calibration was performed according to the manufacturer’s recommendations, with 100 nm polystyrene beads used for verification of optical focus. EVs were analyzed in light-scattering mode using the 488 nm laser. Data acquisition was performed at a shutter setting of 100, camera sensitivity of 78, and 30 frames/s. For post-acquisition analysis, the particle detection settings were defined as an area range of 10–1000 and a minimum brightness of 30. Prior to measurement, EV preparations were diluted with particle-free 0.05 M PBS (pH 7.2) to obtain a particle concentration appropriate for NTA. Three measurements were performed for each sample, with data collected from up to 11 positions.

### 2.11. Quantification of Extracellular Vesicle Protein and Lipid Content

The concentration of protein in EV isolates was measured in technical triplicate using the Bradford protein assay [33]. BSA was used to generate the calibration curve and protein concentrations were determined from the standard curve. The lipid content of EV isolates was evaluated by colorimetric sulfo-phospho-vanillin assay using cholesterol as the standard [34]. Cholesterol standards (25–200 μg/mL) were produced in chloroform. Standards and samples (70 µL) were transferred to microcentrifuge tubes and the solvent was evaporated by heating at 90 °C with the lids open. Subsequently dried cholesterol standards were reconstituted in 50 µL PBS and applied immediately to tubes containing dried lipid samples (50 µL EV solution). After incubation at 90 °C for 20 min, 250 µL of concentrated sulfuric acid were added to each tube. The reaction mixtures were then transferred to a 96-well microplate (220 µL/well) and cooled to room temperature. Then, 110 µL of vanillin reagent (0.2 mg/mL in 17% phosphoric acid) was added and samples were incubated for 10 min before measuring absorbance at 540 nm. The lipid content was measured in terms of cholesterol equivalents with the use of the calibration curve. All lipid measurements were performed in technical duplicate.

### 2.12. Atomic Force Microscopy (AFM)

The EV morphology was examined by atomic force microscopy (AFM) using a NanoScope 3D instrument (Veeco Digital Instruments). Imaging was performed with etched silicon cantilevers having spring constants in the range of 20–80 N/m. Prior to sample deposition, mica substrates were freshly cleaved using adhesive tape to obtain a clean and smooth surface. A small volume of the EV suspension was applied onto the mica and left to dry under ambient conditions. The acquired AFM images were processed and analyzed using Nanoscope software (version 1.40r1).

### 2.13. Flow Cytometry

EV surface markers were analyzed by a bead-based flow cytometry technique. Latex beads (3 µm, Sigma-Aldrich) were diluted in PBS to a final concentration of 1% and the EV sample was added to the bead suspension and incubated overnight at 4 °C with gentle shaking to allow EV adsorption to the bead surface. After incubation, the supernatant was removed and the beads were pelleted by centrifugation at 13,000× *g* for 5 min before being rinsed twice with PBS and then incubated with glycine solution for 30 min. The beads were centrifuged again and further blocked with freshly made 5% (*w*/*v*) skim milk in PBS for extra 30 min. The excess blocking solution was washed off the beads three to four times with PBS until the supernatant was clear. For immunostaining, individual bead suspensions were diluted 1:5 in 1% bovine serum albumin (BSA) in PBS and treated with anti-CD9 (clone MM2/57), anti-CD63 (clone H5C6), and anti-CD81 (clone 5A6). After antibody incubation, the beads were washed with PBS to remove unbound antibodies and examined by a BD FACSCalibur flow cytometer (BD Biosciences, Franklin Lakes, NJ, USA) with a 488 nm blue laser.

### 2.14. Reusability of VHH-Functionalized Magnetite Nanoparticles

The reusability of the developed VHH-functionalized magnetite solid phase was evaluated by performing five consecutive EV isolations using five 330 µL aliquots of the same PFP sample obtained from a healthy donor and the same VHH-functionalized magnetite nanoparticles. After each isolation cycle, the nanoparticles were regenerated by washing three times with PBS and subsequently reused for the next isolation cycle. The reusability of the isolation procedure was assessed by determining the total protein amount and total particle recovery of the isolated EVs.

### 2.15. Effect of Plasma Input Volume on EV Isolation

To evaluate EV isolation from different plasma input volumes and determine the lowest tested volume from which EVs could be detected, EV yields from five different plasma volumes, 25, 50, 100, 200, and 330 µL, were compared. All isolations were performed with the same immunoaffinity protocol, under the same experimental conditions. Obtained isolates were characterized by determining protein content and particle concentration.

### 2.16. Statistical Analysis

Statistical analysis was performed using GraphPad Prism version 10.6.1 (GraphPad Software, Boston, MA, USA). Data are presented as mean ± standard deviation (SD), as indicated in the corresponding figure captions. For the reusability experiment, differences in protein amounts among the five consecutive isolation cycles were analyzed using Welch’s one-way ANOVA followed by Dunnett’s T3 multiple comparisons test. A *p*-value ≤ 0.05 was considered statistically significant.

## 3. Results

### 3.1. Characterization of the Magnetite Particles

**Figure 2:**
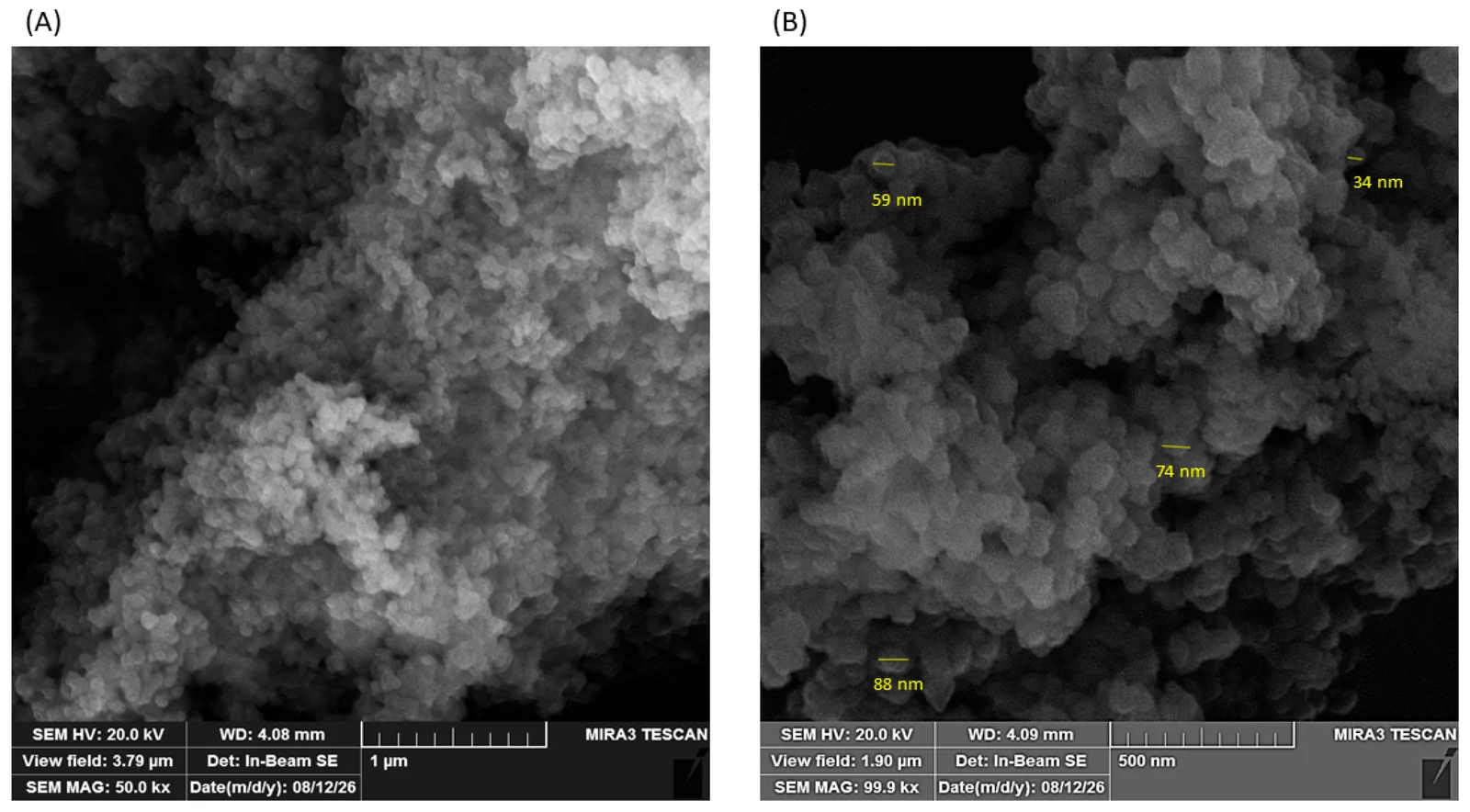
SEM images of magnetite particles at different magnifications: (**A**) 50,000× and (**B**) 100,000×.

The morphology of the synthesized magnetite particles was observed by SEM. As shown in Figure 2, the particles exhibited an approximately spherical morphology, with sizes ranging from approximately 30 to 100 nm. Pronounced agglomeration was also observed. At higher magnification, the agglomerates appeared to be composed of smaller particles.

**Figure 3:**
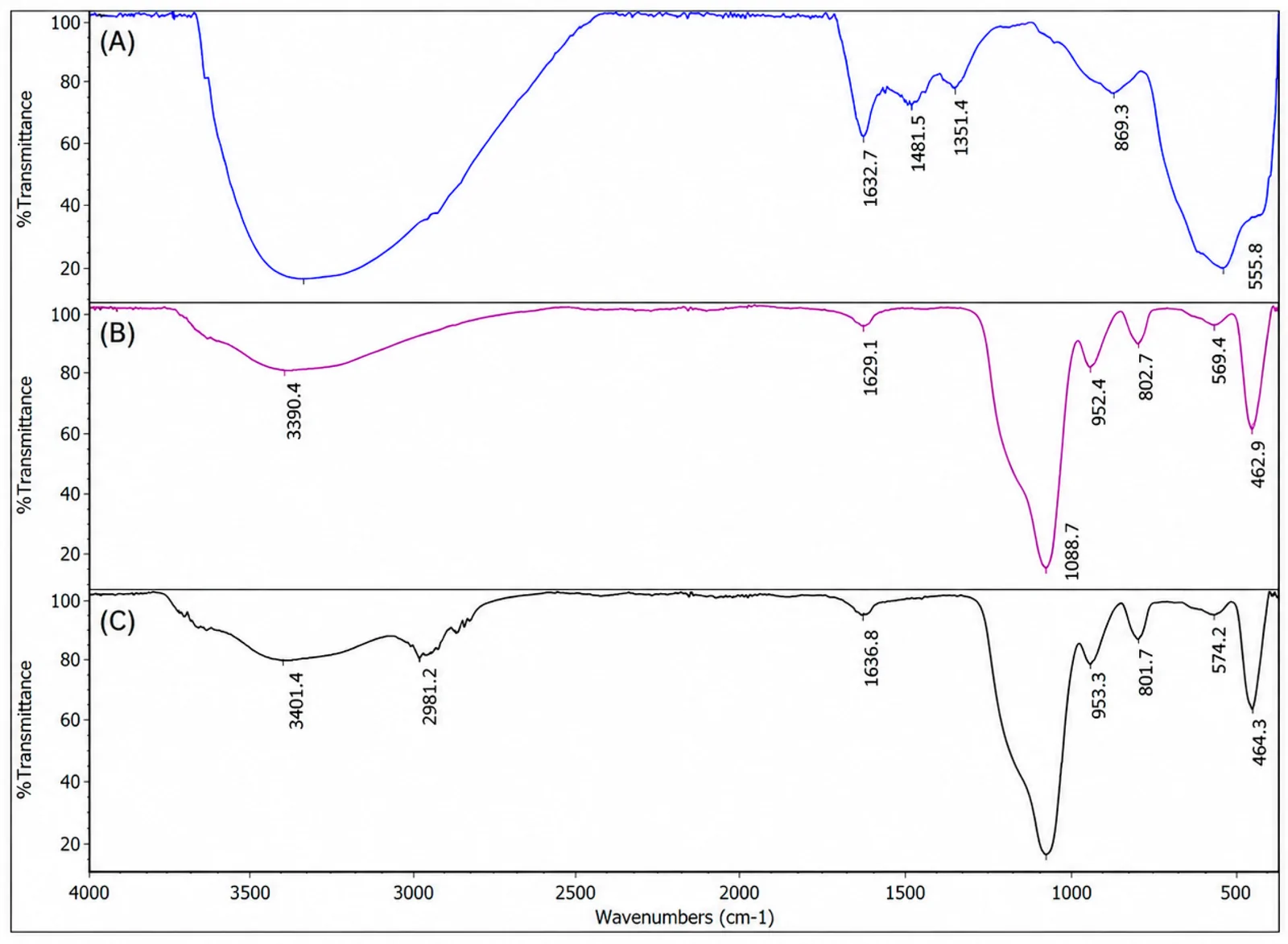
FTIR spectra of magnetite nanoparticles at different stages of surface modification: (**A**) bare magnetite nanoparticles, (**B**) silica-coated magnetite nanoparticles, and (**C**) APTES-functionalized silica-coated magnetite nanoparticles.

The chemical composition of the magnetite nanoparticles was followed during surface modification by FTIR spectroscopy (Figure 3). The spectrum of bare magnetite nanoparticles showed a characteristic absorption band at 555.8 cm^−1^, attributed to Fe–O stretching vibrations. Following the silica coating procedure, characteristic bands were observed at 1088.7 and 802.7 cm^−1^, attributed to Si–O–Si vibrations, together with a band at 952.4 cm^−1^ assigned to Si–OH groups. Following APTES functionalization, a new absorption band at 2981.2 cm^−1^ was observed and attributed to C–H stretching vibrations of the propyl groups of APTES.

### 3.2. Binding Capacity of Functionalized Magnetite Nanoparticles

**Figure 4:**
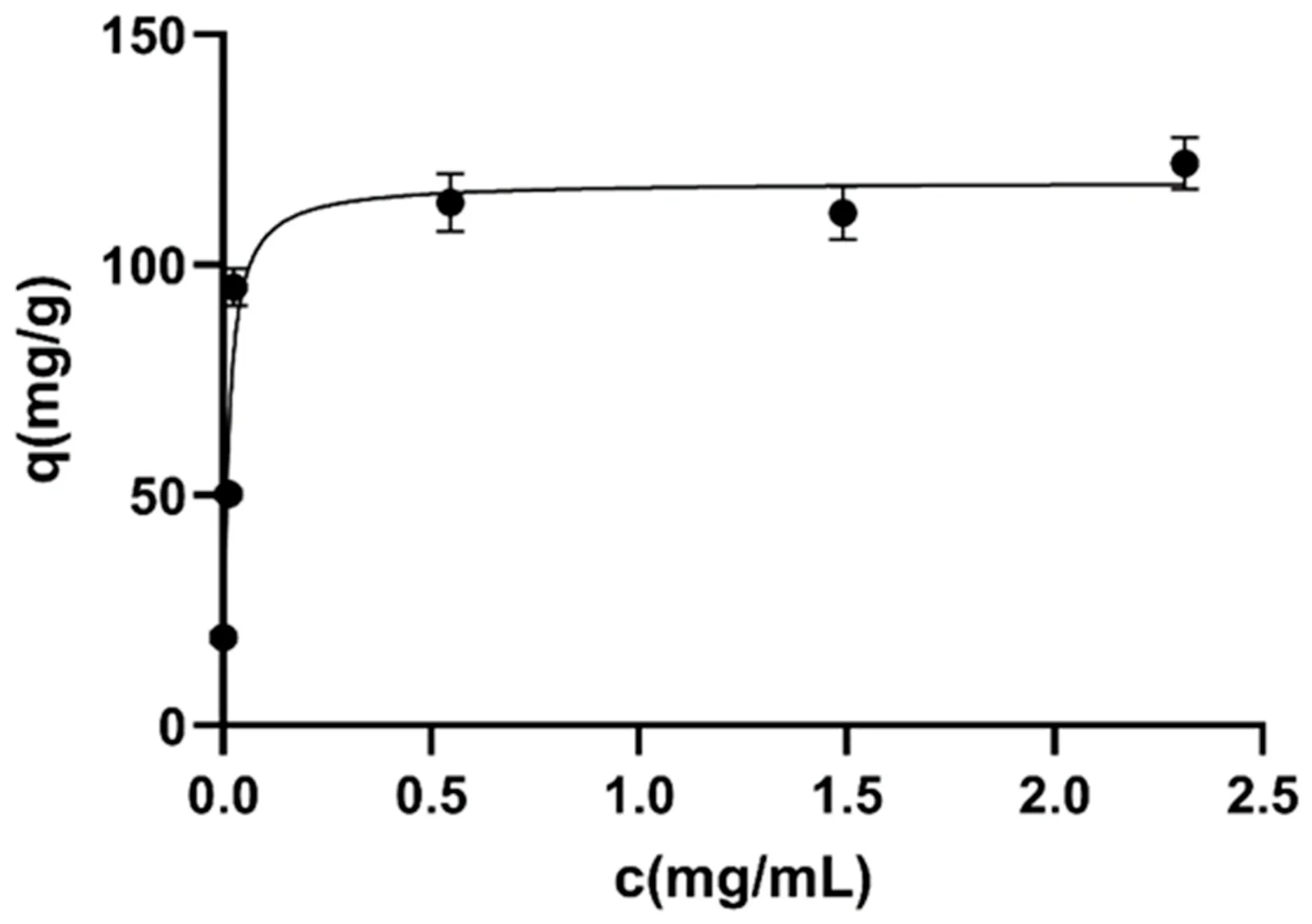
Langmuir adsorption isotherm of BSA on aldehyde-functionalized magnetite nanoparticles. Static binding capacity was quantified using BSA as a model protein. The amount of protein bound per unit mass of dry nanoparticles (q, mg/g) was plotted as a function of the equilibrium concentration of unbound protein after 24 h of incubation, determined using the Bradford protein assay, and fitted using the Langmuir adsorption model.

The protein-binding capacity of the aldehyde-functionalized magnetite nanoparticles was evaluated using BSA as a model protein, and the experimental data were analyzed using the Langmuir adsorption model. The obtained binding isotherm is shown in Figure 4. The binding capacity increased with increasing equilibrium protein concentration until a plateau was reached, indicating saturation of the available binding sites. The maximum binding capacity (*Qmax*) was estimated to be 118.2 mg/g of dry nanoparticles, with an equilibrium dissociation constant (*Kd*) of 0.0111 mg/mL. The Langmuir model adequately described the experimental data (R^2^ = 0.893).

### 3.3. VHH Immobilization on Functionalized Magnetite Nanoparticles

Based on the binding capacity determined using BSA as a model protein, a protein amount below the estimated maximum binding capacity was selected for VHH immobilization. The efficiency of VHH immobilization onto the functionalized magnetite nanoparticles was assessed by quantifying the unbound protein remaining in the supernatant after immobilization using the Bradford protein assay. No residual protein was quantified in the supernatant, indicating that the amount of unbound VHH was below the detection limit of the Bradford assay. 

### 3.4. Quantification and Size Distribution of Isolated EVs

**Figure 5:**
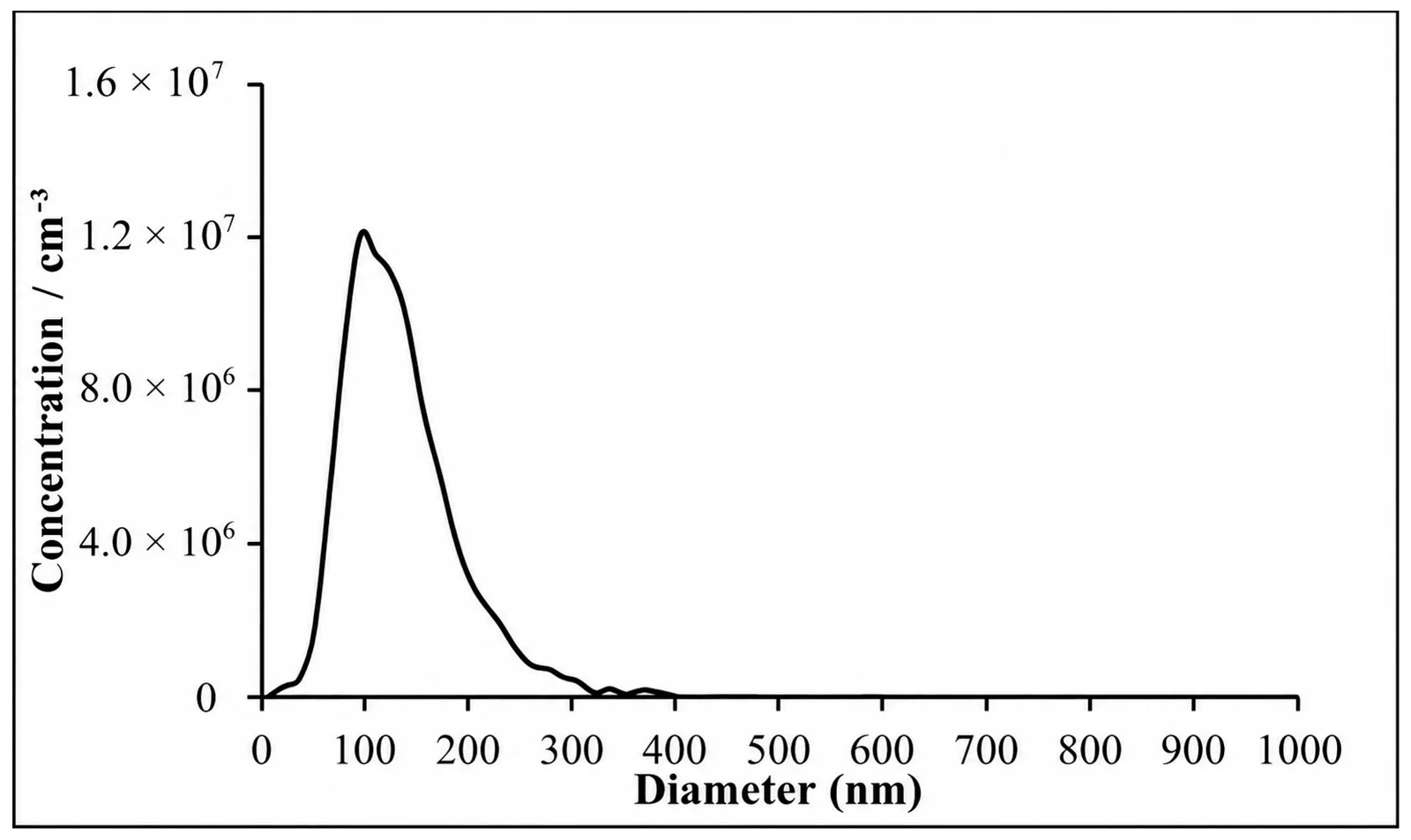
Size distribution of isolated EVs determined by NTA.

The isolated EVs were characterized by determining their protein and lipid contents, as well as particle concentration and size distribution. Starting from 330 µL of plasma, EVs were recovered in a final volume of 1 mL. Protein quantification using the Bradford protein assay yielded a concentration of 0.340 mg/mL, with a total recovered protein amount of 0.340 mg. Protein was below the detection limit of the Bradford assay in the eluate obtained using magnetite nanoparticles without immobilized VHH–eGFP. Lipid content, determined by the sulfo-phospho-vanillin (SPV) assay, was 0.115 mg/mL, corresponding to a recovered lipid amount of 0.115 mg. The calculated protein-to-lipid (P/L) ratio was 2.96. Concentration and size distribution of the isolated extracellular vesicles were further evaluated by NTA (Figure 5). The median particle diameter was 124 nm, which is consistent with the expected EV size, while the measured particle concentration was 6.9 × 10^9^ particles/mL, equivalent to a total recovery of 6.9 × 10^9^ particles.

### 3.5. Flow Cytometry Characterization of Isolated EVs

**Figure 6:**
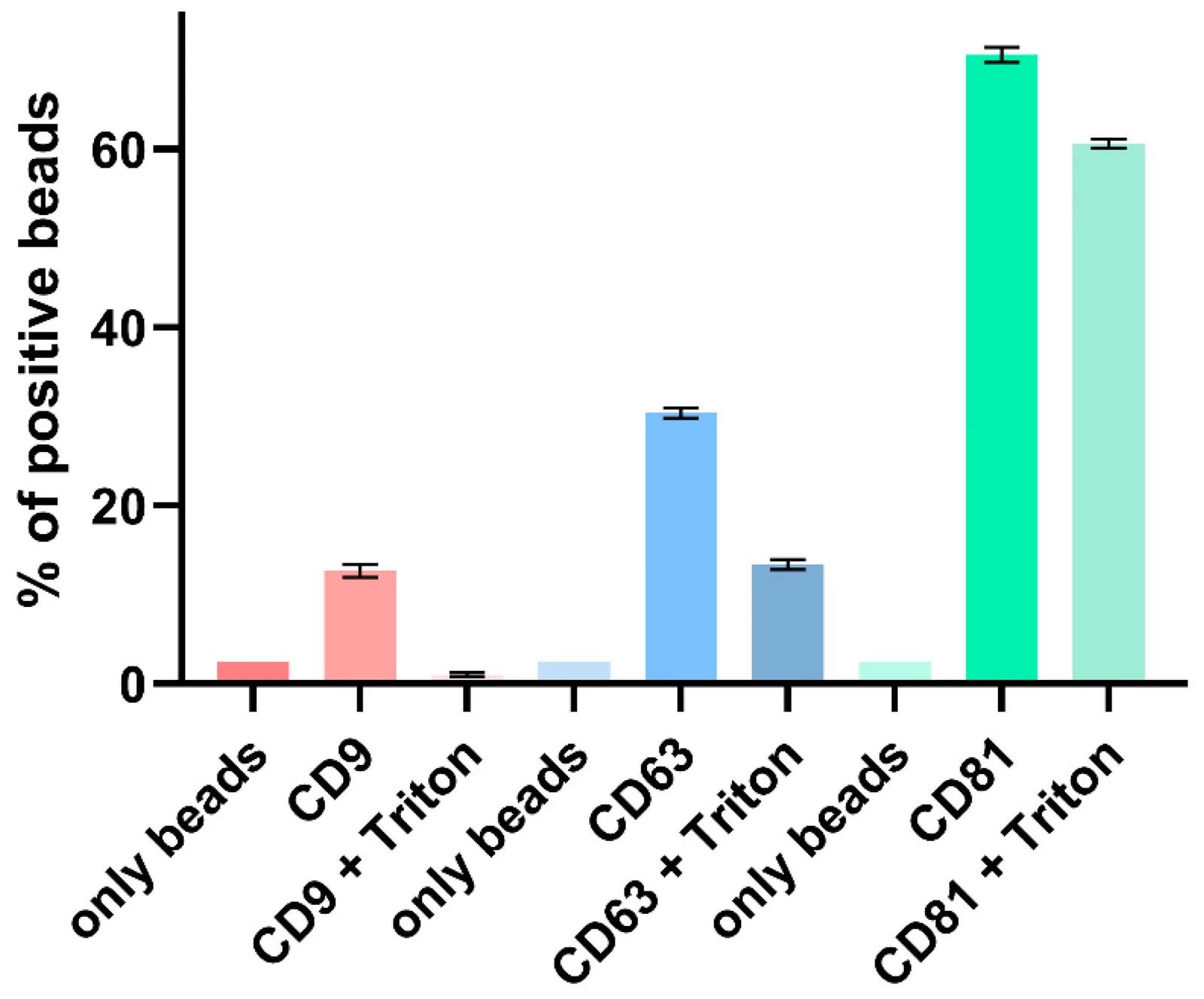
Flow cytometric characterization of isolated EVs. Expression of the EV-associated markers CD9, CD63, and CD81 before and after Triton X-100 treatment. Data are presented as mean ± SD of technical duplicates (n = 2).

To evaluate the presence of the EV-associated tetraspanins CD9, CD63, and CD81 on the isolated particles, flow cytometry was performed. As shown in Figure 6, all three markers showed a higher percentage of positive beads compared to the corresponding bead-only controls, confirming the presence of EV-associated surface proteins. Among the analyzed markers, CD81 displayed the highest percentage of positive beads, followed by CD63 and CD9. Following antibody staining, the samples were treated with Triton X-100 and the change in fluorescence signal was monitored by flow cytometry. The percentage of positive beads decreased after detergent treatment for all three markers, indicating the disruption of the vesicular membrane and supporting that the detected tetraspanins were associated with EV membranes.

### 3.6. Atomic Force Microscopy Characterization of Isolated EVs

**Figure 7:**
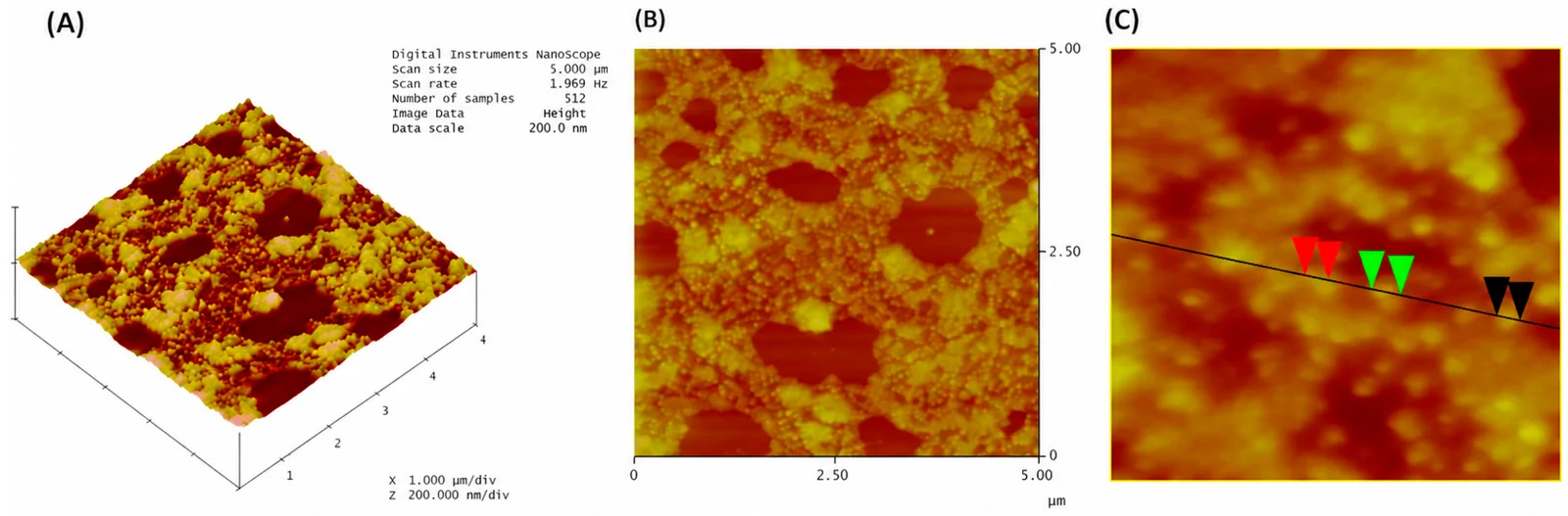
Atomic force microscopy characterization of isolated EVs. Representative 3D (**A**) and 2D (**B**) topographical images and the corresponding cross-sectional profile (**C**) used for vesicle size determination.

The morphology of isolated EVs was further characterized by atomic force microscopy (AFM). Representative 3D (Figure 7A) and 2D (Figure 7B) topographical images revealed the presence of mostly spherical nanosized particles. The dimensions of representative vesicles were estimated from the cross-sectional profile shown in Figure 7C. The measured diameters were 50.8 nm (black), 70.3 nm (green) and 48.8 nm (red), confirming that the isolated particles were in the expected size range of EVs.

### 3.7. Reusability of EV Isolation

**Figure 8:**
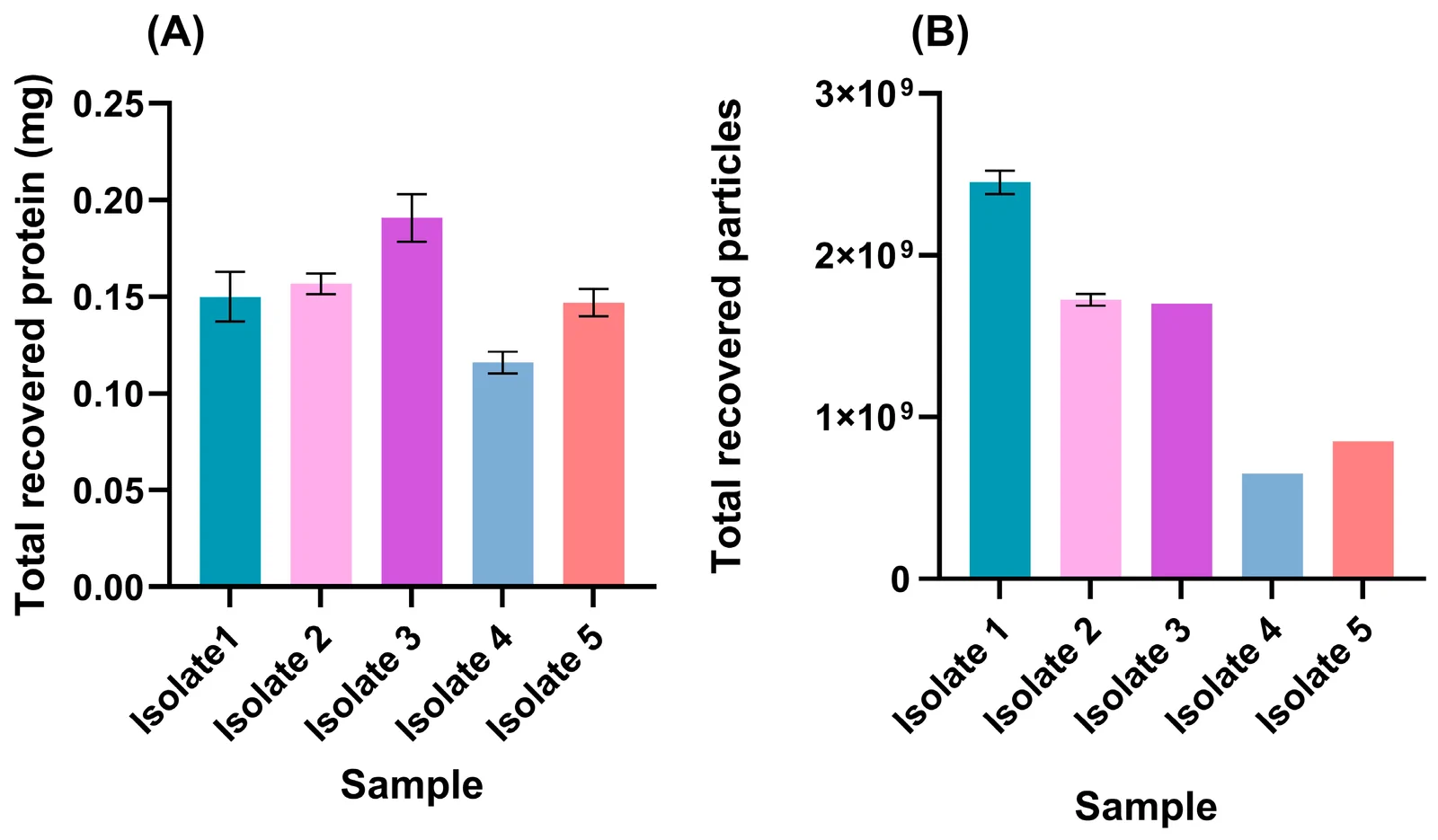
Reusability of EV isolation using VHH-functionalized magnetite nanoparticles. (**A**) Protein amount and (**B**) total particle recovery determined by NTA after five consecutive isolation cycles. Bars represent mean values from technical duplicates (n = 2), and error bars indicate standard deviation (SD). Statistical differences in protein amount were evaluated using Welch’s one-way ANOVA followed by Dunnett’s T3 multiple comparisons test. A significance level of *p* ≤ 0.05 was considered statistically significant. ns: not significant.

Five consecutive EV isolations were performed using the same batch of VHH-functionalized magnetite nanoparticles to test the reusability of the developed magnetic solid phase. Protein and particle concentrations obtained by NTA after each isolation cycle are shown in Figure 8. The total protein amount of the isolated EVs was 0.153 ± 0.026 mg, with only moderate variation between the consecutive isolation cycles (Figure 8A). Likewise, the NTA revealed a median total particle recovery of (1.48 ± 0.73) × 10^9^ particles, while all isolates were within the same order of magnitude (Figure 8B).

### 3.8. Evaluation of EV Isolation from Different Plasma Volumes

**Figure 9:**
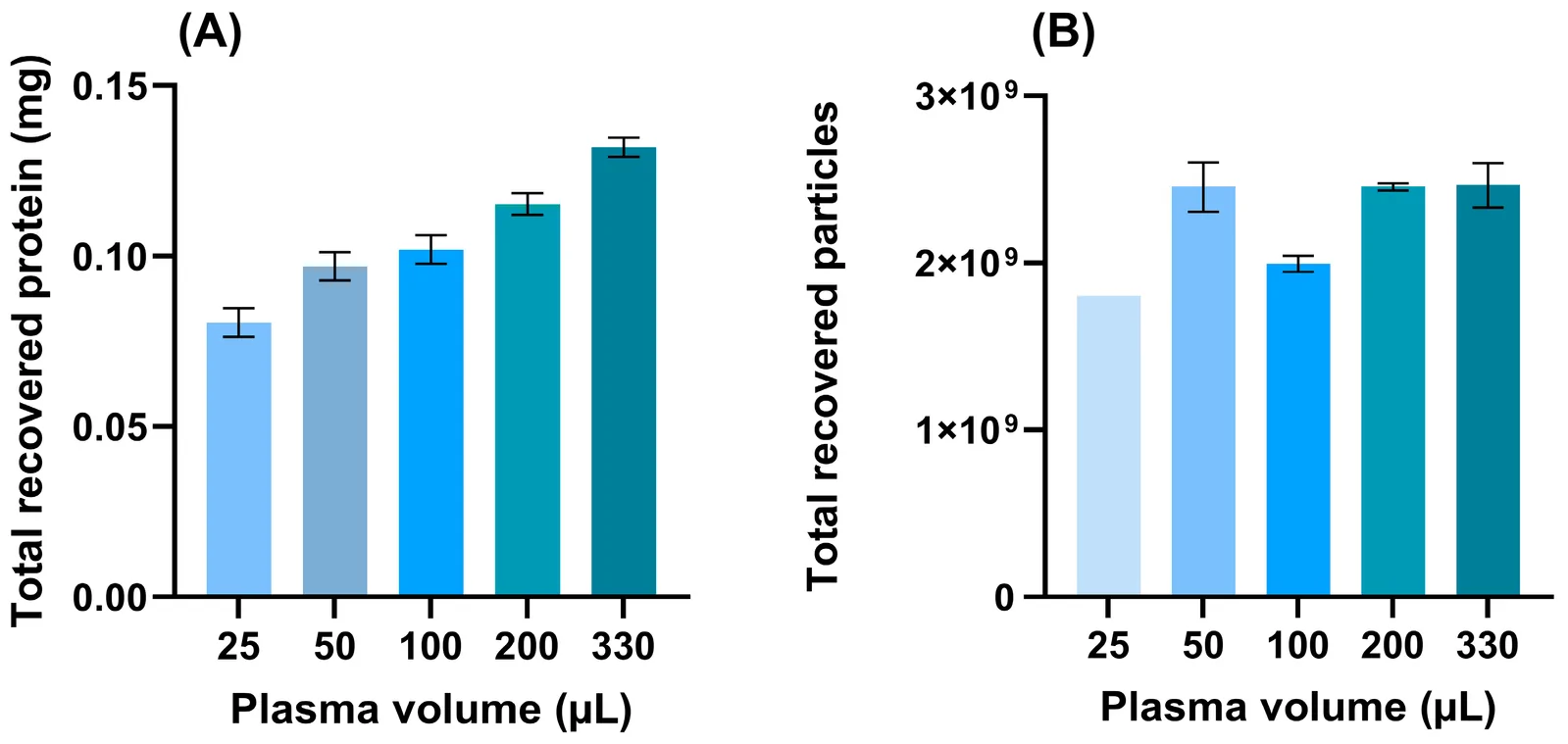
Effect of the initial plasma volume on EV isolation. (**A**) Protein amount and (**B**) total particle recovery of EV isolates obtained from different starting plasma volumes. Bars represent mean values from technical triplicates (n = 3), and error bars indicate standard deviation (SD).

EV isolation was evaluated using plasma input volumes of 25, 50, 100, 200, and 330 µL to determine the lowest tested volume from which EVs could be detected. The total protein amount of the EV isolates increased with increasing plasma volume, as shown in Figure 9. NTA revealed total particle recoveries in the order of 10^9^ particles across all tested plasma volumes, with particles detected even at the lowest plasma volume.

## 4. Discussion

EVs have attracted considerable attention in recent years due to their involvement in a wide range of biological processes. Consequently, considerable effort has been directed toward developing reliable and reproducible approaches for EV isolation and characterization. Differential ultracentrifugation is widely used for EV isolation [35]. However, it is labor-intensive and time-consuming and requires specialized equipment, while the high centrifugal forces applied may contribute to vesicle aggregation and recovery of non-vesicular material [36,37]. A number of alternative approaches have consequently been developed, including size-exclusion chromatography, ultrafiltration, polymer-based precipitation, and microfluidic platforms [38]. Each of these methods represents a compromise between yield, purity, processing time, scalability, and cost. Among the available approaches, immunoaffinity-based isolation provides a particularly high degree of selectivity because it relies on the specific interaction between affinity ligands and surface markers expressed on EVs [13]. Conventional monoclonal antibodies have been widely employed as capture molecules for this purpose. More recently, VHH nanobodies have emerged as an attractive alternative owing to their small size, high stability, favorable biochemical properties, and high target specificity [39]. Moreover, VHHs selected from naïve libraries have already been successfully applied for the selective capture of EVs [40].

Magnetic nanoparticles represent highly attractive solid supports for affinity-based EV isolation because they enable rapid magnetic separation while providing a large surface area for biomolecule immobilization [18,41]. Their surfaces can also be readily modified with different functional groups, allowing the development of versatile affinity platforms [42,43]. In this study, an immunoaffinity platform based on silica-coated magnetic nanoparticles functionalized with a mixture of five VHH-eGFP constructs was developed for the isolation of EVs from human plasma. A mixture of five previously selected EV-binding VHHs were used, with the aim of broadening recognition across heterogeneous EV populations. Silica-coated magnetic nanoparticles were selected as the solid support because the silica shell provides a chemically stable and readily functionalized surface [44,45,46]. The silica beads offer a chemically stable surface for further functionalization, which allows modification with APTES and subsequent covalent immobilization of the VHH-eGFP mediated by glutaraldehyde. This functionalization strategy was selected with the aim of promoting stable ligand attachment during washing, elution, and potential reuse of the affinity material [46]. SEM analysis revealed that the magnetite particles possessed an approximately spherical morphology and sizes ranging from 30 to 100 nm. Similar morphologies and sizes of Fe_3_O_4_ nanoparticles were previously reported [47]. Agglomeration was also observed in the present study and is commonly reported for Fe_3_O_4_ nanoparticles and is attributed to high surface energy and strong interparticle interactions [48,49,50]. Further, FTIR analysis supported the successful stepwise surface modification of the magnetite nanoparticles. For bare magnetite, the Fe–O band was in agreement with the expected Fe–O vibrations of Fe_3_O_4_ nanoparticles [51,52]. Following the silica coating procedure, characteristic Si–O–Si and Si–OH bands were observed, supporting the presence of silica on the surface of the Fe_3_O_4_ nanoparticles [53,54]. However, FTIR analysis alone does not provide direct evidence of the uniformity or morphology of the silica layer. Also, the appearance of C–H stretching band after APTES functionalization was attributed to the stretching vibrations of –CH_2_– groups of APTES [54]. Protein-binding studies using BSA as a model protein were performed to evaluate the protein immobilization capacity of the activated nanoparticles. The experimental data were well described by the Langmuir adsorption model, consistent with predominantly monolayer adsorption onto relatively uniform binding sites. *Qmax* was 118 mg g^−1^, indicating a high density of available surface sites on the activated nanoparticles for protein immobilization. Nevertheless, excessively high ligand densities can potentially compromise functional binding through steric hindrance or unfavorable ligand orientation, emphasizing that maximal protein loading does not necessarily correspond to maximal EV-capture efficiency. No VHH was detected in the supernatant after immobilization, indicating that the amount of unbound protein was below the detection limit of the Bradford assay. However, this indirect assessment does not provide direct evidence of covalent VHH attachment or preservation of VHH functional activity after immobilization. The ability of the functionalized nanoparticles to enrich EV-associated particles from plasma was therefore evaluated experimentally. Specifically, the performance of the immunoaffinity material was exploited for the isolation of EVs from human plasma. Starting from 330 µL of plasma, the resulting EV-enriched preparations contained a total protein amount of 0.340 mg and a total lipid amount of 0.115 mg, corresponding to a protein-to-lipid ratio of 2.96. Nanoparticle tracking analysis revealed a total recovery of 6.9 × 10^9^ particles and a median particle diameter of 124 nm, values consistent with the expected range for EV-containing preparations. However, as particle size alone is not sufficient to confirm EV identity, the isolated particles were further assessed using complementary characterization methods. Flow cytometry analysis confirmed the presence of EV-associated tetraspanins CD9, CD63, and CD81, with all three markers showing increased fluorescence relative to the bead-only control. CD81 exhibited the strongest signal, followed by CD63 and CD9, consistent with the heterogeneous distribution of tetraspanins among EV populations. Importantly, treatment with Triton X-100 resulted in a marked decrease in fluorescence intensity, supporting the membrane-enclosed vesicular nature of the detected particles. The residual CD81 signal observed after Triton X-100 treatment may reflect incomplete detergent-mediated disruption and/or retention of CD81-containing components on the bead surface. However, the present experiment does not allow the underlying mechanism to be determined, and this result should therefore be interpreted with caution. Negative EV markers were not assessed in the present study; therefore, the presence of co-isolated non-EV components cannot be completely excluded. Additional analysis of EV-negative and plasma-associated contaminant markers will be important in further evaluation of the purity of the isolated preparations. Atomic force microscopy provided additional evidence regarding particle morphology. AFM images revealed nanoscale structures with dimensions of approximately 50–70 nm, within the expected size range of small EVs.

The developed platform also demonstrated reusability over five consecutive isolation cycles. EV preparations obtained in successive isolation cycles showed total recovered protein amounts ranging from 0.117 to 0.190 mg, while total particle recoveries measured by NTA ranged from 0.65 × 10^9^ to 2.45 × 10^9^ particles. These findings demonstrate that the affinity material could be regenerated and reused for EV isolation over five consecutive cycles.

Importantly, EVs could also be isolated from plasma volumes as low as 25 μL. Although protein yield increased with increasing input volume, total particle recoveries remained in the order of 10^9^ particles across all tested plasma volumes, demonstrating that EVs could be detected even from limited sample volumes. This feature may be particularly advantageous for applications in which sample availability is restricted. The functional activity of the isolated EV preparations was not evaluated in the present proof-of-concept study and should be addressed in future downstream functional assays.

## 5. Conclusions

In conclusion, VHH-functionalized silica-coated magnetite nanoparticles were developed and applied for the immunoaffinity isolation of EVs from human plasma. The functionalized nanoparticles enabled the enrichment of membrane-enclosed, tetraspanin-positive particles from plasma. The combination of magnetic separation with VHH-based recognition provided a rapid and adaptable platform that could be reused over repeated isolation cycles and was suitable for EV isolation from low plasma volumes.

These findings support the potential of VHH-functionalized magnetite nanoparticles as an adaptable platform for immunoaffinity-based EV isolation. The modular nature of the platform also provides opportunities to tailor the VHH composition toward specific EV subpopulations or disease-associated surface markers. Further optimization of ligand density, immobilization chemistry, elution, and regeneration conditions may improve EV recovery and selectivity. In addition, long-term stability, extended reusability, performance across larger and clinically diverse sample cohorts, and direct comparison with established EV isolation methods should be investigated to further assess the platform’s suitability for translational and diagnostic applications.

## Data Availability

Data can be found within the article.
